# Association of Concomitant Gestational Hypertensive Disorders and Gestational Diabetes With Cardiovascular Disease

**DOI:** 10.1001/jamanetworkopen.2022.43618

**Published:** 2022-11-23

**Authors:** Justin B. Echouffo Tcheugui, Jun Guan, Longdi Fu, Ravi Retnakaran, Baiju R. Shah

**Affiliations:** 1 Division of Endocrinology, Diabetes & Metabolism, Department of Medicine, Johns Hopkins University, Baltimore, Maryland; 2 Institute for Clinical Evaluative Sciences, Toronto, Ontario, Canada; 3 Leadership Sinai Centre for Diabetes, Mount Sinai Hospital, Toronto, Ontario, Canada; 4 Lunenfeld-Tanenbaum Research Institute, Mount Sinai Hospital, Toronto, Ontario, Canada; 5 Division of Endocrinology, University of Toronto, Toronto, Ontario, Canada; 6 Department of Medicine, Sunnybrook Health Sciences Centre, Toronto, Ontario, Canada; 7 Institute for Health Policy Management and Evaluation, University of Toronto, Toronto, Ontario, Canada

## Abstract

**Importance:**

Accruing evidence suggests that gestational hypertensive disorders (GHTD) and gestational diabetes (GD) are each associated with an increased risk of cardiovascular disease (CVD). However, the extent to which the co-occurrence of GHTD and GD is associated with the risk of CVD remains largely unknown.

**Objective:**

To estimate the individual and joint associations of GHTD and GD with incident CVD.

**Design, Setting, and Participants:**

This population-based cohort study used the Ministry of Health and Long-Term Care of Ontario (Canada) health care administrative databases. All women in Ontario with a GHTD and/or GD diagnosis, and a live-birth singleton delivery between July 1, 2007, and March 31, 2018, were considered for inclusion. Women with pregravid diabetes, hypertension, or cardiovascular disease were excluded. Statistical analysis was performed from November 2021 to September 2022.

**Exposures:**

GD and/or GHTD, defined using diagnosis coding.

**Main Outcomes and Measures:**

Individual and joint associations of GHTD and GD with incident CVD (including a composite of myocardial infarction, acute coronary syndrome, stroke, coronary artery bypass grafting, percutaneous coronary intervention, or carotid endarterectomy), estimated using Cox regression models, adjusting for relevant cardiometabolic risk factors. The follow-up extended from the index pregnancy until March 31, 2020.

**Results:**

Among 886 295 eligible women (mean [SD] age, 30 [5.6] years; 43 861 [4.9%] with isolated GHTD, 54 061 [6.1%] with isolated GD, and 4975 [0.6%] with GHTD and GD), there were 1999 CVD events over 12 years of follow-up. In the early postpartum phase (first 5 years post partum), there was no association of co-occurrence of GTHD and GD (adjusted hazard ratio [aHR], 1.42, 95% CI, 0.78-2.58) or GD alone (aHR, 0.80; 95% CI, 0.60-1.06) with CVD; there was an association between isolated GTHD and incident CVD compared with no GTHD and no GD (aHR, 1.90; 95% CI, 1.51-2.35). In the late postpartum period (after the initial 5 years post partum), compared with no GD and no GHTD, isolated GHTD (aHR, 1.41, 95% CI, 1.12-1.76) and co-occurrence of GHTD and GD (aHR, 2.43, 95% CI, 1.60-3.67) were each associated with a higher risk of incident CVD. There was no association between isolated GD and incident CVD.

**Conclusions and Relevance:**

In this cohort study, GHTD was associated with a high risk of CVD post partum, and the co-occurrence of GD and GHTD was associated with a much greater postpartum CVD risk. These findings suggest that CVD preventive care is particularly needed in the aftermath of combined GD and GHTD.

## Key Points

**Question:**

What is the association of the concomitant occurrence of gestational diabetes (GD) and gestational hypertensive disorder (GHTD) with the incidence of cardiovascular diseases (CVD)?

**Findings:**

In a cohort study of 886 295 women, compared with no GD and no GHTD, the co-occurrence of GHTD and GD was associated with a 2.4-fold higher risk of incident CVD. A risk that is significantly higher than that of the CVD risk associated with each of these conditions in isolation.

**Meaning:**

These findings suggest that the co-occurrence of GD and GHTD is associated with a high postpartum CVD risk.

## Teaser

This cohort study estimates the individual and joint associations of gestational hypertensive disorders (GHTD) and gestational diabetes (GD) with cardiovascular disease (CVD).

## Introduction

Cardiovascular disease (CVD) is common^1^ and increasingly frequent among young individuals, including women of childbearing age.^2^ Accumulating evidence suggests that gestational hypertensive disorders (GHTD) (a forerunner of hypertension^3^)^4,5,6,7^ and gestational diabetes (GD) (a precursor of diabetes^8^)^9^ are each associated with a high risk of developing CVD in the years after pregnancy. However, there is a dearth of studies on the joint influence of GHTD and GD on the incidence of cardiovascular events. The extant study that has examined the joint association of GHTD and GD with outcomes^10^ did not include cardiovascular outcomes separately from the mortality outcome. The existing studies showing a significant association of the co-occurrence of GD and GHTD with an adverse cardiometabolic risk factor profile^11^ and with poor neonatal outcomes,^12^ compared with isolated GD or isolated GHTD, suggest that the joint occurrence of GD and GHTD would be similarly associated with poor postpartum cardiovascular outcomes.

Using data from the health care administrative databases from the Ontario Ministry of Health and Long-Term Care (MOHLTC) in Canada, we sought to examine the individual and conjoint associations of GHTD and GD with the risks of CVD. We hypothesized that the combined presence of GHTD and GD would be associated with a higher risk of CVD postpartum than each of these individual conditions, in which case the identification of GHTD and GD in young women could help in the stratification of the risk of future CVD.

## Methods

### Study Population

The use of data in this study was authorized under section 45 of Ontario’s Personal Health Information Protection Act and hence did not require review by a research ethics board. We followed the Strengthening the Reporting of Observational Studies in Epidemiology (STROBE) reporting guideline for cohort studies.

Our sample of participants consisted of all the women in Ontario, the most populous province in Canada. These women have their health data captured in the health care administrative databases from the MOHLTC. The databases include the Canadian Institute for Health Information (CIHI) Discharge Abstract Database from all hospitalizations and Same Day Surgery from day surgery institutions in Ontario, the Ontario Health Insurance Plan (OHIP) physician service claims for reimbursement for virtually all physician consultations, procedures, and visits; and the Registered Persons Database for demographic information for all residents eligible for health care in Ontario. The Ontario Hypertension Database is a validated registry of physician-diagnosed nongestational hypertension that is derived using these data.^13^ The Ontario Diabetes Database is a validated registry of physician-diagnosed nongestational diabetes that is derived using these data as well as prescription records in the Ontario Drug Benefit (ODB) database.^14^ The MOMBABY database is derived from hospitalization data, and links hospitalization records of delivering mothers with their newborn babies. Individuals are linked between data sources through a unique and reproducibly encrypted health card number.

In the present study, we included women with a live-birth singleton delivery between July 1, 2007, and March 31, 2018. We excluded women with a history of diabetes, a history of hypertension, or a history of CVD prior to the index pregnancy, which were ascertained using the relevant *International Statistical Classification of Diseases and Related Health Problems, Tenth Revision, Canada (ICD-10-CA)* codes. For women with multiple deliveries, only the first delivery was considered in our investigation. The additional criteria of exclusion from the study are detailed in eFigure in Supplement 1.

### Ascertainment of Gestational Hypertensive Disorder and Gestational Diabetes

GHTD was ascertained using the *ICD-10-CA* codes O13, O14, and O15 (gestational hypertension was defined using the O13 code, and eclampsia/preeclampsia was defined using the O14 and O15 codes) in the hospital delivery records done within 24 weeks prior to delivery. GD was identified using the *ICD-10-CA* codes E1 and O24 in the hospital records at index pregnancy or physician billings with a diagnosis *ICD-9* code 250 claimed within 90 days prior to delivery. Using the GHTD and GD status of each woman, we classified them as having neither of the conditions, either, or both. Although our ascertainment did not directly rely on glucose measurements, in Canada, screening and testing for GD is typically performed between 24 and 28 weeks of gestation, using a 2-step approach, which involves screening using a 50-g glucose challenge test (GCT).^15^ When GCT is positive (140 to 199 mg/dL [to convert mg/dL to mmol/L, multiply by 0.0555]), a 75-g OGTT is administered (cutoff values: fasting greater than or equal to95 mg/dL [5.3 mmol/L]; 1 hour greater than or equal to 190 mg/dL [10.6 mmol]; 2 hours greater than or equal to 162 mg/dL [9.0 mmol/L]). GD is defined as 1 or more abnormal OGTT values or a GCT greater than or equal to 200mg/dL (11.1 mmol/L).^15^

### Cardiovascular Outcomes

The outcome was incident CVD defined as a composite of hospitalization for myocardial infarction, acute coronary syndrome, stroke, coronary artery bypass grafting, percutaneous coronary intervention, or carotid endarterectomy. The women were followed from the index pregnancy until cardiovascular disease event, death, migration, or March 31, 2020, whichever came first. The CVD events were identified through linkage with databases including a hospital discharge database for clinical diagnoses and procedures, and day surgery institutions for percutaneous intervention (PCI), from the index gestation until March 31, 2020. The *ICD-10 *codes I20 to I25 were used for coronary heart disease, and the *ICD-10* codes I60 to I69, G08, G46, and H34 for cerebrovascular disease. In addition, we also used the procedure codes for coronary artery bypass graft and carotid endarterectomy (codes shown in eTable 1 in Supplement 1).

### Covariates

The covariates considered in our analyses included age at index delivery, socioeconomic status (ascertained ecologically based on neighborhood household income quintile), rurality of residence (ascertained using the Rurality Index of Ontario),^16^ parity, preterm delivery, chronic kidney disease (defined using a previously validated algorithm^17^), GHTD at prior pregnancy, GD at prior pregnancy, preexisting circulatory disease (other than the outcomes of interest), postpartum progression to diabetes, and postpartum development of hypertension.

### Statistical Analyses

The baseline characteristics of the study population were presented by categories of GHTD and GD, including 4 groups: (1) no GD and no-GHTD, (2) no-GD and GHTD, (3) no-GHTD and GD, and (4) GD and GHTD. Continuous variables were presented as mean and SD (or median and IQR for variables with skewed distributions), and categorical variables as percentages. The differences in baseline characteristics were tested using χ^2^ tests for categorical variables and appropriate parametric or nonparametric tests for continuous variables.

For incident CVD, we assessed the risk across the exposure categories (with no GD and no-GHTD as the reference), using Cox proportional hazards regression models. The outcome was first compared between the study groups using the log-rank test with Kaplan-Meier curves. Proportionality assumptions were tested by Schoenfeld residuals for both individual covariates and the global Schoenfeld test. This revealed time-dependency in the association of study groups with the CVD outcome. We therefore built piecewise Cox proportional hazards models over 2 periods^18^:– (1) an early postpartum phase (first 5 years postpartum) and a late postpartum phase (after initial 5 years after index delivery). In addition to the aforementioned comparisons, we also conducted a direct comparison between isolated GD (reference) and isolated GHTD, in terms of incident CVD outcome, using similar Cox proportional hazards regression models.

We conducted sequential adjustments. In an initial model (model 1), we adjusted for age at index delivery, socioeconomic status, and parity. In a subsequent model (model 2), we adjusted for model 1 variables, rurality of residence, preterm delivery, chronic kidney disease, GHTD in a previous pregnancy, GD in a previous pregnancy, and preexisting circulatory disease (other than the CVD outcomes of interest). In a final model (model 3), we adjusted for model 2 variables, postpartum development of diabetes (as a time-varying covariate), and postpartum development of hypertension (as a time-varying covariate).

Two-sided *P* < .05 was considered statistically significant. All analyses were done using SAS version 9.4 (SAS Institute) from November 2021 to September 2022.

## Results

The study population consisted of 886 295 women (mean [SD] age, 30 [5.6] years), among whom 43 861 (4.9%) had isolated GHTD, 54 061 (6.1%) had isolated GD, and 4975 (0.6%) had a combination of GD and GTHD. Table 1 shows the baseline characteristics of the study population by GD and/or GHTD status. Women with GD and GHTD were older, less likely to have a premature delivery and prior GHTD, but more likely to have developed postpartum diabetes, and postpartum hypertension. The characteristics of study participants by CVD status at follow-up are also shown in eTable 2 in Supplement 1.

**Table 1.** Baseline Characteristics of Study Participants by Gestational Hypertensive Disorder and/or Gestational Diabetes Status

Characteristics	Participants, No. (%)				*P* value
	GD−, GHTD− (N = 783 398)	GD−, GHTD+ (N = 43 861)	GD+, GHTD− (N = 54 061)	GD+, GHTD+ (N = 4975)	
Age at index pregnancy, mean (SD), y	29.60 (5.54)	29.61 (5.72)	32.20 (5.23)	31.92 (5.75)	<.001
Neighborhood income quintile					
1 (lowest)	177 830 (22.7)	9554 (21.8)	14 765 (27.3)	1342 (27.0)	<.001
2	159 564 (20.4)	9237 (21.1)	11 824 (21.9)	1064 (21.4)	
3	159 424 (20.4)	9208 (21.0)	11 055 (20.4)	1042 (20.9)	
4	159 617 (20.4)	8971 (20.5)	9725 (18.0)	920 (18.5)	
5 (highest)	124 008 (15.8)	6672 (15.2)	6498 (12.0)	573 (11.5)	
Rurality					
Urban	596 194 (76.1)	30 905 (70.5)	46 155 (85.4)	3828 (76.9)	<.001
Semi-urban	130 942 (16.7)	9183 (20.9)	5676 (10.5)	755 (15.2)	
Rural	56 262 (7.2)	3773 (8.6)	2230 (4.1)	392 (7.9)	
No. of previous births					
0	514 315 (65.7)	35 368 (80.6)	32 111 (59.4)	3576 (71.9)	<.001
1	173 642 (22.2)	5428 (12.4)	13 336 (24.7)	800 (16.1)	
≥2	95 441 (12.2)	3065 (7.0)	8614 (15.9)	599 (12.0)	
Preterm delivery (gestational age ≤36 weeks)	42 748 (5.5)	6608 (15.1)	4449 (8.2)	1035 (20.8)	<.001
Chronic kidney disease at baseline	1841 (0.2)	244 (0.6)	158 (0.3)	52 (1.0%)	<.001
Gestational diabetes in a previous pregnancy	5314 (0.7)	258 (0.6)	3375 (6.2)	243 (4.9)	<.001
Gestational hypertension in a previous pregnancy	7303 (0.9)	1984 (4.5)	775 (1.4)	258 (5.2)	<.001
Postpartum diabetes (before CVD)	10 886 (1.4)	1184 (2.7)	9169 (17.0)	1077 (21.6)	<.001
Postpartum hypertension (before CVD)	23 420 (3.0)	6381 (14.5)	3561 (6.6)	1025 (20.6)	<.001
Preexisting circulatory disease	6639 (0.8)	547 (1.2)	466 (0.9)	69 (1.4)	<.001

Abbreviations: CVD, cardiovascular disease; GD, gestational diabetes; GD−, no GD; GD+, GD present; GHTD, gestational hypertensive disorder; GHTD−, no GHTD; GHTD+, GHTD present.

Over the total follow-up period (12.8 years, 7.0 million person-years), there were 1999 CVD events (867 during the first 5 years postpartum and 1162 events during the period subsequent to the first 5 years post index delivery). The Figure shows the Kaplan-Meier cardiovascular disease-free survival curves by study groups across the entire study period.

**Figure.**
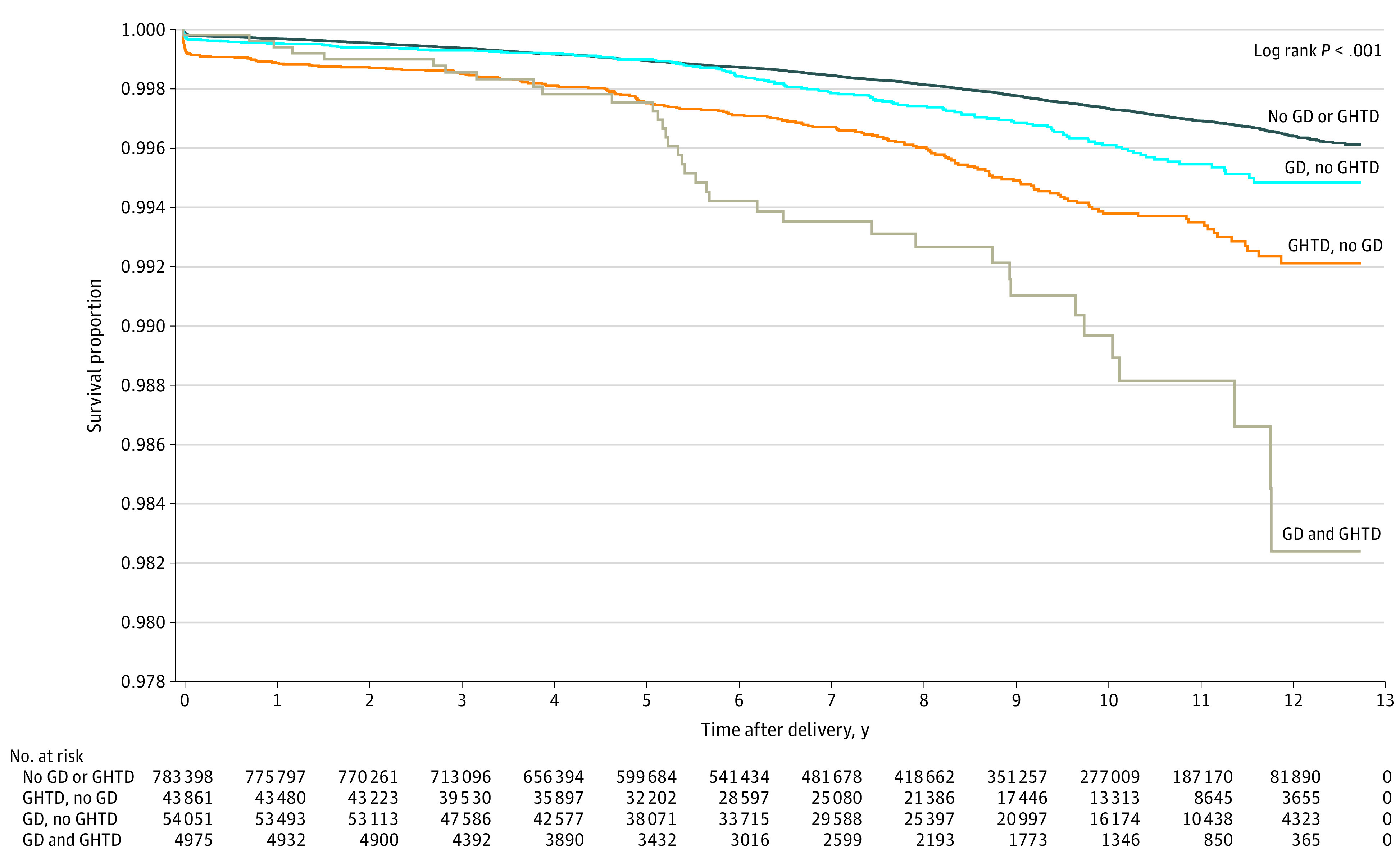
Kaplan-Meier Cardiovascular Disease–Free Survival Curves by Gestational Diabetes (GD) and Gestational Hypertensive Disorder (GHTD) Status, Across the Entire Study Period

### Early Postpartum Stage (First 5 Years After Index Delivery)

During the initial 5 years after index delivery, individuals who experienced both GD and GHTD had the highest incidence of CVD events (highest absolute risk) of all study groups (Table 2). Their relative risk of incident CVD as compared with those without GD or without GHTD was however attenuated with adjustment for confounders such that it was no longer statistically significantly higher. Indeed, after full adjustment for confounders (including postpartum diabetes and postpartum hypertension), the estimate for the association of incident CVD with the co-occurrence of GHTD and GD (adjusted hazard ratio [aHR]: 1.42; 95% CI, 0.78-2.58; *P* = .25) was not significantly different from that of those with no GHTD and no GD (model 3 in Table 2). The corresponding estimate of association with CVD (model 3 in Table 2) was significant for isolated GHTD (aHR, 1.90; 95% CI, 1.53-2.35; *P* < .001) but was not associated with isolated GD (aHR, 0.80; 95% CI, 0.60-1.06; *P* = 0.12).

**Table 2.** Event Rates and Hazard Ratios for the Association of Gestational Hypertensive Disorder and Gestational Diabetes With Incident Cardiovascular Disease

Exposures	Early phase (initial 5 years after index pregnancy)					Late phase (after the initial 5 years following the index delivery)				
	Crude IR per 10 000 PY (95% CI)	Hazard ratio (95% CI)				Crude IR per 10 000 PY (95% CI)	Hazard ratio (95% CI)			
		Unadjusted	Adjusted model 1^a^	Adjusted model 2^b^	Adjusted model 3^c^		Unadjusted	Adjusted Model 1^a^	Adjusted Model 2^b^	Adjusted Model 3^c^
No GHTD and no GD	1.94 (1.80-2.08)	1 [Reference]	1 [Reference]	1 [Reference]	1 [Reference]	3.54 (3.32-3.78)	1 [Reference]	1 [Reference]	1 [Reference]	1 [Reference]
Isolated GD	1.89 (1.42-2.52)	0.99 (0.75-1.31)	0.84 (0.63-1.12)	0.82 (0.61-1.09)	0.80 (0.60-1.06)	6.05 (4.95-7.39)	1.76 (1.42-2.18)	1.50 (1.21-1.87)	1.44 (1.151-1.79)	1.19 (0.95-1.50)
Isolated GTHD	4.04 (3.25-5.02)	2.33 (1.89-2.87)	2.40 (1.95-2.96)	2.13 (1.72-2.63)	1.90 (1.53-2.35)	8.18 (6.78-9.86)	2.10 (1.69-2.60)	2.19 (1.76-2.72)-	1.90 (1.53-2.38)	1.41 (1.12-1.76)
GHTD and GD	4.47 (2.41-8.31)	2.32 (1.28-4.21)	2.04 (1.12-3.70)	1.71 (0.94-3.10)	1.42 (0.78-2.58)	18.89 (12.86-27.74)	5.61 (3.77-8.35)	4.99 (3.34-7.42)	4.05 (2.71-6.05)	2.43 (1.60-3.67)

Abbreviations: GD, gestational diabetes; GHTD, gestational hypertensive disorder; IR, incidence rate; PY, person-years.

^a^ Model 1: adjusted for age, neighborhood income quintile, and parity.

^b^ Model 2: model 1 plus rurality, chronic kidney disease, prior gestational hypertensive disorder, and prior gestational diabetes.

^c^ Model 3: model 1 plus postpartum diabetes and postpartum hypertension.

During the first 5 years after the index delivery, after full adjustment (including postpartum hypertension, and postpartum diabetes) isolated GHTD was associated with a greater risk for incident CVD than isolated GD (aHR, 2.32; 95% CI, 1.62-3.30; *P* < .001) in a direct comparison.

### Late Postpartum Stage (After the Initial 5-Year Period After Index Delivery)

After the initial 5-year period following the index delivery, there were notable differences in absolute risk (incidence rate) of cardiovascular events across the study groups (Table 2). The highest absolute CVD risk was observed among those with concomitant GD and GHTD, which was approximately 2- to 3-fold that of isolated GD or isolated GHTD.

Individuals who experienced both GD and GHTD had a 2.4-fold higher relative risk of CVD (aHR: 2.43; 95% CI, 1.60-3.67; *P* < .001) compared to those without GHTD nor GD (Table 2, model 3), after full adjustment for covariates (including postpartum diabetes and postpartum hypertension). The corresponding estimates for the association of CVD with isolated GHTD (aHR, 1.41; 95% CI, 1.12, 1.76; *P* = .003) and with isolated GD (aHR, 1.19; 95% CI, 0.95-1.55; *P* = .13), using the no GD and no GHTD groups as the reference category, were lower (model 3 in Table 2). A direct comparison showed that isolated GHTD and isolated GD (reference) had similar risks of incident CVD after full adjustment (aHR, 1.12; 95% CI, 0.81-1.56; *P* = .49), after the initial 5-year period post index delivery.

## Discussion

In this large population-based cohort study, extended follow-up after delivery found that while GHTD and GD would each be individually associated with a high risk of CVD, women who experienced both GHTD and GD during a pregnancy were ultimately at significantly higher absolute and relative risk of CVD, as compared to those without either of these conditions. Over time, this risk became more pronounced than that observed among women with each of these conditions separately. The observed association with concomitant GHTD and GD was present even after accounting for postpartum diabetes and postpartum hypertension.

To our knowledge, this study is the largest in which the joint association of GHTD and GD with postpartum CVD has been examined. Our results corroborate those reported in a prior study on the deleterious cardiovascular impact of the co-occurrence of GHTD and GD. The latter study was however smaller in size compared to ours, did not examine CVD separately from all-cause mortality,^10^ and did not comprehensively account for potential confounding factors (eg, postpartum diabetes, and postpartum hypertension).^10^ Another small study showed that the joint occurrence of GD and GHTD was associated with a more adverse postpartum cardiometabolic risk factor profile than isolated GD or GHTD, but it did not include cardiovascular outcomes.^11^ Our findings on the individual and separate associations of GHTD and GD with the future risk of CVD are also congruent with the results from prior studies showing a link between GHTD and elevated risk of postpartum CVD,^4,19,20^ between GD and higher risk of CVD postpartum,^9,21^ as well as between GHTD or GD and subclinical CVD outcomes.^22,23^ Our observations extend the literature on the links between GD, GHTD and CVD, by providing additional evidence on the concomitant associations of GHTD and GD with CVD incidence postpartum. The combination of GHD and GD may represent a more severe form of maladaptive pregnancy changes that substantially contribute to future CVD.

Our findings on the extent of CVD risk associated with concomitance of GHTD and GD have potential practical implications. These suggest that systematic detection of GHTD and GD during pregnancy is important for long-term prediction of future CVD in the postpartum. The relevance of such screening is illustrated by the rising burden CVD in the younger population^2^; the risk of CVD related to GD and GTHD,^4,9,19,20,21^ and the increasing prevalence of both GTHD and GD in Canada^24^ and the US.^25,26^ Many women (>80%) will undergo pregnancy during their lifetime,^27^ thus screening for GHTD and GD during pregnancy should lead to a more effective surveillance and modification of CVD risk factors among young women in the postpartum period. This prevention opportunity is congruent with the recommendations of the American Heart Association^28^ and American College of Obstetricians and Gynecologists.^29,30^

The mechanistic pathways linking GD and GHTD to a heightened CVD risk are incompletely understood. Insulin resistance could be a common underlying pathway linking both GHTD and GD to CVD.^31,32^ Studies have identified pregnancy-related changes common to GHTD and GD, which include endothelial dysfunction (lower flow-mediated dilation), angiogenenic imbalance (high soluble fms-like tyrosine kinase 1 [sFlt-1] and/or low placental growth factor [PGF]), increased oxidative stress, and dyslipidemia.^33^ Postpartum incidence of type 2 diabetes or hypertension among women with GD and GHTD may mediate the occurrence of CVD, although only partially.^34,35^ Indeed, in our analyses, adjusting for postpartum diabetes and postpartum hypertension affected the magnitude of the association of GD/GHTD with subsequent CVD. GD and GHTD may reflect a latent underlying and intrinsic high-risk phenotype characterized by cardiometabolic dysregulation, and hence the heighten CVD risk over time.^36,37^ It is also possible that the postpartum CVD risks of CVD related to GD and GHTD are affected by the underlying changes in behavioral risk factors (mainly physical activity and dietary intake) over time. Thus, partly explaining the differential CVD risks observed in the immediate vs distant postpartum periods.

### Limitations and Strengths

There are limitations to our study. First, some cardiovascular risk factors, are not captured in administrative databases used in our investigation, thus the possibility of residual confounding. These include biological (eg, lipid levels and body mass index) and behavioral (eg, smoking, alcohol use, dietary intake, and physical activity) factors. It is important to indicate that extant evidence suggests that women with GHTD or GD have an adverse CVD risk factor profile irrespective of body mass index before,^38,39,40,41^ and after pregnancy.^42,43,44,45,46^ Women with and without GD or GHTD may not also differ in the extent of changes in adiposity in the aftermath of pregnancy.^47^ Second, we did not use glucose data to define GD, but GD diagnosis from administrative data has been shown to be reliable and valid.^48^ The definitions of GHTD based on *ICD* codes have also been shown to be valid.^49^ We did not have enough power to examine the subtypes of GHTD (preeclampsia/eclampsia and gestational hypertension) individually. We used a composite cardiovascular outcome, which limits our ability to understand the associations of GD and/or GHTD with specific individual cardiovascular outcomes, and thus which outcome may drive the observed association. Lastly, we lacked data on the use of cardioprotective medications in the postpartum period.

Our study has strengths that include the assessment of a large population-based cohort of women, from a health care system in which there is systematic assessment for both GHTD and GD among pregnant women, and that allows post-partum follow-up for CVD including data on both events and relevant procedures. We conducted a rigorous adjustment including known cardiovascular risk factors, post-partum diabetes and post-partum hypertension, as well as pregnancy-specific factors such as prior histories of GD and GHTD.

## Conclusions

This cohort study found that over time, co-occurrence of GD and GHTD was associated with a much greater postpartum CVD risk than the individual conditions. The systematic identification of both GHTD and GD in obstetrical practice offers an opportunity for a more effective CVD prevention among young women of childbearing age.

## Notes

Supplement 1. **eFigure.** Process of Selection of the Study Participants **eTable 1.** Canadian Classification of Diagnostic, Therapeutic, and Surgical Procedures (CCP) Codes for Percutaneous Intervention (PCI), Coronary Artery Bypass Graft (CABG), and Carotid Endarterectomy **eTable 2.** Baseline Characteristics of Study Participants by Cardiovascular Disease Status

Supplement 2.
Data Sharing Statement
