# Supplementary material for: Association of Concomitant Gestational Hypertensive Disorders and Gestational Diabetes With Cardiovascular Disease
Source: JAMA Netw Open. 2022 Nov 23;5(11):e2243618. doi: 10.1001/jamanetworkopen.2022.43618 (PMC9685489; doi:10.1001/jamanetworkopen.2022.43618)
Supplement: Supplement 1. — eFigure. Process of Selection of the Study Participants eTable 1. Canadian Classification of Diagnostic, Therapeutic, and Surgical Procedures (CCP) Codes for Percutaneous Intervention (PCI), Coronary Artery Bypass Graft (CABG), and Carotid Endarterectomy eTable 2. Baseline Characteristics of Study Participants by Cardiovascular Disease Status [file jamanetwopen-e2243618-s001.pdf]

Supplemental Online Content

Echouffo-Tcheugui JB, Guan J, Fu L, Retnakaran R, Shah BR. Association of concomitant gestational hypertensive disorders and gestational diabetes with cardiovascular disease. *JAMA Netw Open.* 2022;5(11):e2243618. doi:10.1001/jamanetworkopen.2022.43618

eFigure. Process of Selection of the Study Participants

eTable 1. Canadian Classification of Diagnostic, Therapeutic, and Surgical Procedures (CCP) Codes for Percutaneous Intervention (PCI), Coronary Artery Bypass Graft (CABG), and Carotid Endarterectomy

eTable 2. Baseline Characteristics of Study Participants by Cardiovascular Disease Status

This supplemental material has been provided by the authors to give readers additional information about their work.

© 2022 Echouffo-Tcheugui JB et al. *JAMA Network Open.*

eFigure 1. Process of selection of the study participants

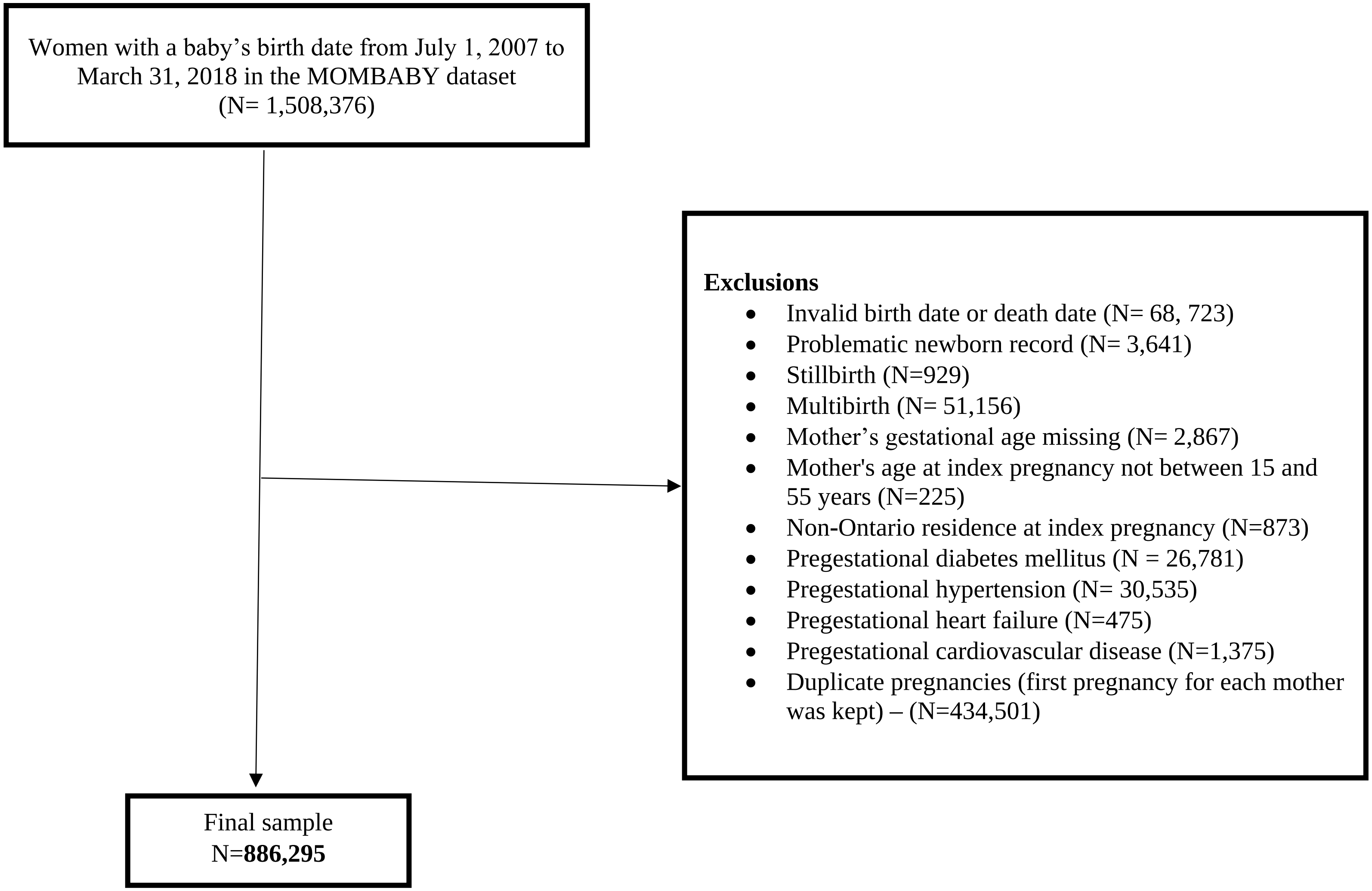

The ICES-derived MOMBABY database contains linked Canadian Institute for Health Information's Discharge Abstract Database (CIHI-DAD) maternal and newborn hospital birth abstracts, as well as unlinked maternal and newborn birth abstracts (henceforth known as orphan birth records).

© 2022 Echouffo-Tcheugui JB et al. *JAMA Network Open.*

eTable 1. Canadian Classification of Diagnostic, Therapeutic, and Surgical Procedures (CCP) codes for percutaneous intervention (PCI), coronary artery bypass graft (CABG), and carotid endarterectomy

Procedure	Canadian Classification of Health Intervention (CCI)	Canadian Classification of Diagnostic, Therapeutic, and Surgical Procedures (CCP)	The description of CCP codes
PCI	1IJ50	4802	Percutaneous transluminal coronary angioplasty (PTCA) without mention of thrombolytic agent
	1IJ54	480	Removal of coronary artery obstruction
		4801	Removal of coronary artery obstruction
		4803	Percutaneous transluminal coronary angioplasty (PTCA)
		4805	Intracoronary artery thrombolytic infusion
		4809	Other Removal of coronary artery obstruction
	1IJ55	4899	Other operation on vessels of heart nec
	1IJ57	4802	Percutaneous transluminal coronary angioplasty (PTCA) without mention of thrombolytic agent
		4804	Open chest coronary artery angioplasty
CABG	1IJ76	481	Bypass anastomosis for heart revascularization
		4811	Aorto-coronary bypass for heart revascularization, unqualified
		4812	Aorto-coronary bypass for one coronary artery
		4813	Aorto-coronary bypass for two coronary arteries
		4814	Aorto-coronary bypass for three coronary arteries
		4815	Aorto-coronary bypass for four or more coronary arteries
		4816	Single (internal) mammary -coronary artery bypass
		4817	Double (internal) mammary -coronary artery bypass
		4819	Other bypass anastomosis for heart revascularization
	1IJ80	482	Heart revascularization by arterial implant
		4899	Other operations on vessels of the heart nec
Carotid endarterectomy	1JE55	5149	Other revision of vascular procedure
	1JE57	5012	Endarterectomy of other vessels of head and neck
		5032	Resection of other vessels of head and neck with replacement

© 2022 Echouffo-Tcheugui JB et al. *JAMA Network Open.*

eTable 2. Baseline characteristics of study participants by cardiovascular disease status

**Characteristics**	**No CVD**	**CVD**	***P-value***
	N=884,296	N=1,999	
GDM and GHTD status			
GDM-GHTN-	781,767 (88.4)	1,631 (81.6)	<.001
GDM-GHTN+	43,671 (4.9)	190 (9.5%)	
GDM+GHTN-	53,919 (6.1)	142 (7.1%)	
GDM+GHTN+	4,939 (0.6)	36 (1.8%)	
Age at index date, years mean (SD)	29.77 (5.57)	31.40 (5.89)	<.001
Neighborhood Income Quintile			
1 (lowest)	202,996 (23.0)	495 (24.8)	0.009
2	181,254 (20.5)	435 (21.8)	
3	180,336 (20.4)	393 (19.7)	
4	178,835 (20.2)	398 (19.9)	
5 (highest)	137,486 (15.5)	265 (13.3)	
Rurality			
Urban	675,605 (76.4)	1,477 (73.9)	0.013
Semi-Urban	146,203 (16.5)	353 (17.7)	
Rural	62,488 (7.1)	169 (8.5)	
Parity			
0	584,358 (66.1)	1,012 (50.6)	<.001
1	192,668 (21.8)	538 (26.9)	
≥2	107,270 (12.1)	449 (22.5)	
Preterm Delivery (gestational age ≤ 36 weeks)	54,610 (6.2)	230 (11.5)	<.001
Chronic kidney disease at baseline	2,280 (0.3)	15 (0.8)	<.001
Gestational diabetes in a previous pregnancy	9,135 (1.0)	55 (2.8)	<.001
Gestational hypertension in a previous pregnancy	10,260 (1.2)	60 (3.0)	<.001
Postpartum diabetes (before CVD)	22,238 (2.5)	78 (3.9)	<.001
Postpartum hypertension (before CVD)	34,159 (3.9)	228 (11.4)	<.001
Pre-existing circulatory disease	7,648 (0.9)	73 (3.7)	<.001

Data are mean (standard deviation) or N (%)
CVD: Cardiovascular disease, GHTD: gestational hypertensive disorder, GDM: gestational diabetes.
GHTD- = no-GHTD, GHTD+ = GHTD, GDM - = no-GDM, GDM + = GDM

© 2022 Echouffo-Tcheugui JB et al. *JAMA Network Open.*
