# Supplementary material for: Association of Concomitant Gestational Hypertensive Disorders and Gestational Diabetes With Cardiovascular Disease
Source: JAMA Netw Open. 2022 Nov 23;5(11):e2243618. doi: 10.1001/jamanetworkopen.2022.43618 (PMC9685489; doi:10.1001/jamanetworkopen.2022.43618)
Supplement: Supplement 2. — Data Sharing Statement [file jamanetwopen-e2243618-s002.pdf]

Data Sharing Statement

Echouffo-Tcheugui JB, Guan J, Fu L, Retnakaran R, Shah BR. Association of concomitant gestational hypertensive disorders and gestational diabetes with cardiovascular disease. *JAMA Netw Open*. 2022;5(11):e2243618. doi:10.1001/jamanetworkopen.2022.43618

Data
Data available: No
